# Motion-corrected compressed-sensing enables robust spiral first-pass perfusion imaging with whole heart coverage

**DOI:** 10.1186/1532-429X-16-S1-O81

**Published:** 2014-01-16

**Authors:** Yang Yang, Xiao Chen, Frederick H Epstein, Craig H Meyer, Sujith Kuruvilla, Christopher M Kramer, Michael Salerno

**Affiliations:** 1 Biomedical Engineering, University of Virginia, Charlottesville, Virginia, USA; 2 Medicine, University of Virginia, Charlottesville, Virginia, USA; 3 Radiology, University of Virginia, Charlottesville, Virginia, USA

## Background

First-pass perfusion imaging using CMR is an important tool for diagnosing coronary artery disease (CAD), but most clinical techniques are limited in their spatial coverage. While compressed-sensing (CS) holds promise for highly accelerated perfusion spiral imaging, CS techniques suffer from blurring artifacts in the setting of respiratory motion. Spiral pulse sequences have multiple advantages for myocardial perfusion imaging including high acquisition efficiency, high signal to noise (SNR) and robustness to motion and for CS including a relatively incoherent aliasing pattern. Thus, we develop a whole heart spiral first-pass perfusion sequence combined with robust motion-correction.

## Methods

Eight subjects undergoing clinical scans were recruited for rest perfusion studies using an accelerated 4× spiral perfusion sequence with whole heart coverage. The sequence parameters included: 2 variable density interleaves, 6.1 ms readout per interleaf, TE 1.0 ms, TR 9 ms, TI 80 ms, FA 45°, FOV 340 mm^2^, in-plane resolution of ~2 mm, 6 ~ 10 slices to cover the whole heart. All perfusion images were acquired on a 1.5T Siemens Avanto scanner during infusion of 0.1 mmol/kg of Gd-DTPA. The images were reconstructed by direct reconstruction with zero padding (DC), L1-SPIRiT using finite time difference as the sparsity transform, and Block LOw-rank Sparsity with Motion guidance compressed sensing (BLOSM). The images were evaluated for quality and blurring by two experienced cardiologists.

## Results

**Figure 1:**
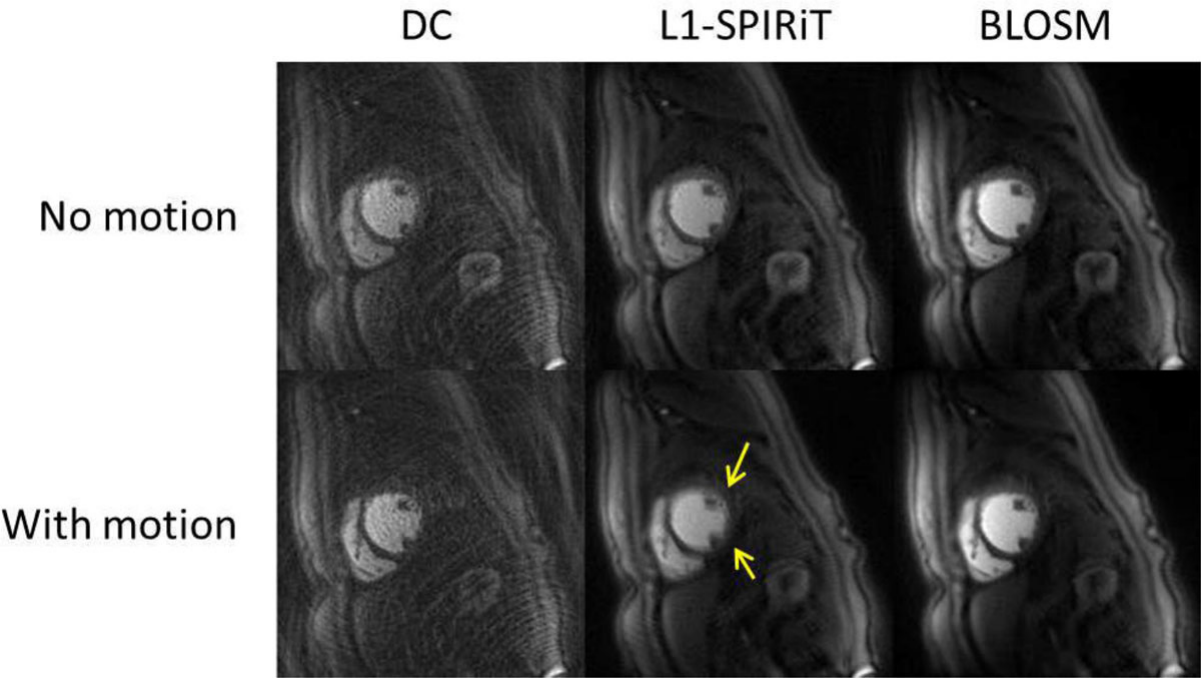
**Perfusion images reconstructed by direct recon with DC, L1-SPIRiT and BLOSM with (top) and without(bottom) motion**.

**Figure 2:**
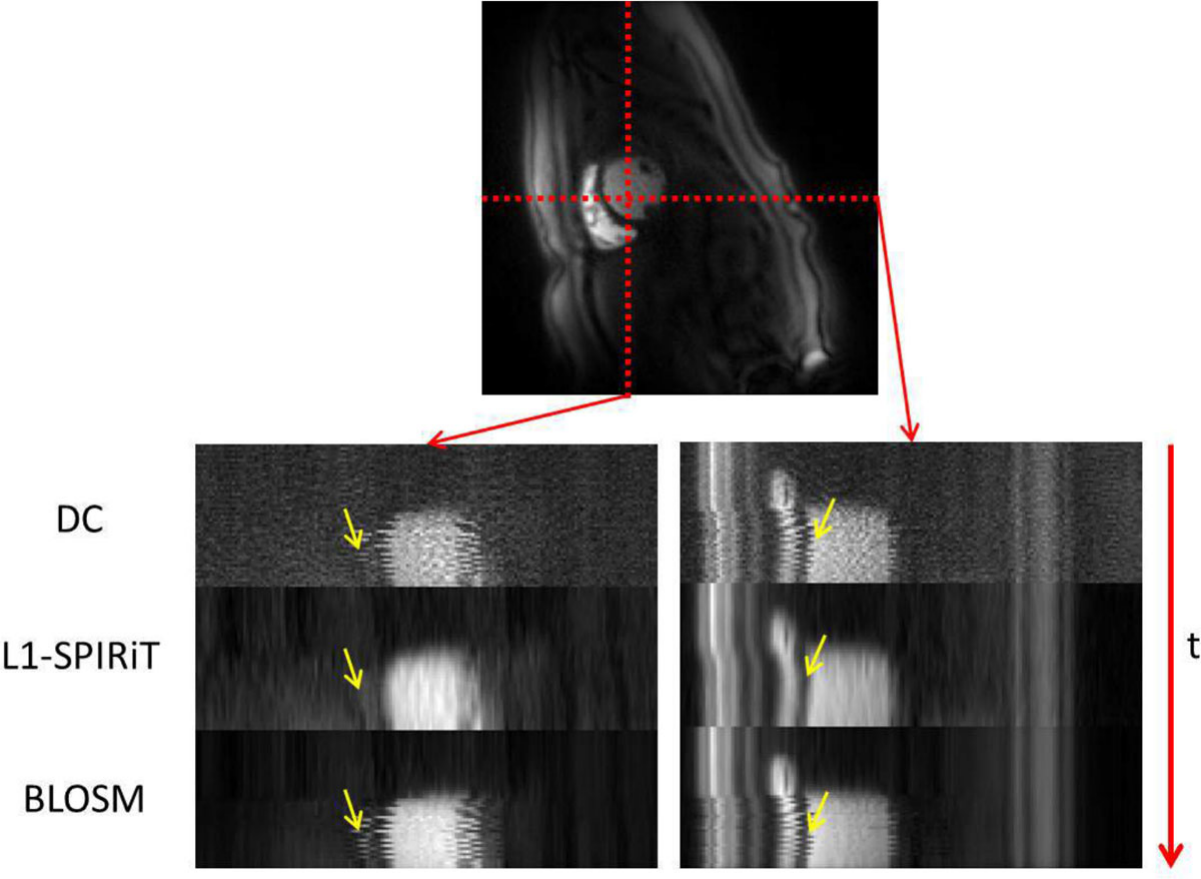
**Time profile curve from DC, L1-SPIRiT, and BLOSM reconstructed images**.

Figure 1 shows a comparison of perfusion data reconstructed by DC, L1-SPIRiT and BLOSM with (top) and without (bottom) motion from the same subject at different time points. In the absence of motion, both the L1-SPIRiT and BLOSM images have high SNR and image quality without motion artifacts. However, in the presence of motion, the L1-SPIRiT reconstructed images suffer from temporal blurring artifacts (arrows in Figure 1.) as finite difference was imposed on the non-registered images as the sparsity transform. The BLOSM technique imposes sparsity of the tracked blocks which preserves edge information with minimal blurring. Figure 2 shows the spatial-temporal profiles across one subject from the three reconstruction methods. The DC profile shows sharp edges but with high artifacts and noise level. The L1-SPIRIT profile demonstrates high SNR but severe temporal blurring(highlighted by the yellow arrow). The BLOSM images show preserved temporal fidelity and high SNR even in the presence of significant respiratory motion. One cardiologist commented that for all of the data set, the BLOSM had better image quality and less blurring than L1-SPIRiT while the other cardiologist rated BLOSM better for 7 of 8 data sets.

## Conclusions

We demonstrate the feasibility of whole heart spiral first pass perfusion imaging using BLOSM to perform robust CS even in the setting of significant respiratory motion. Further validation will be required in patients undergoing vasodilator stress CMR.

## Funding

K23 HL112910, R01 HL079110.

